# Frustration of Negative Capacitance in Al_2_O_3_/BaTiO_3_ Bilayer Structure

**DOI:** 10.1038/srep19039

**Published:** 2016-01-08

**Authors:** Yu Jin Kim, Min Hyuk Park, Young Hwan Lee, Han Joon Kim, Woojin Jeon, Taehwan Moon, Keum Do Kim, Doo Seok Jeong, Hiroyuki Yamada, Cheol Seong Hwang

**Affiliations:** 1 Department of Materials Science & Engineering and Inter-university Semiconductor Research Center, College of Engineering, Seoul National University, Seoul 151-744, Republic of Korea; 2 Electronic Materials Center, Korea Institute of Science and Technology, Hwarangno 14-gil 5, Seongbuk-gu, 136-791 Seoul, Republic of Korea; 3 National Institute of Advanced Industrial Science and Technology (AIST) and JST, PRESTO, Higashi 1-1-1, Tsukuba, Ibaraki 305-8562, Japan

## Abstract

Enhancement of capacitance by negative capacitance (NC) effect in a dielectric/ferroelectric (DE/FE) stacked film is gaining a greater interest. While the previous theory on NC effect was based on the Landau-Ginzburg-Devonshire theory, this work adopted a modified formalism to incorporate the depolarization effect to describe the energy of the general DE/FE system. The model predicted that the SrTiO_3_/BaTiO_3_ system will show a capacitance boost effect. It was also predicted that the 5 nm-thick Al_2_O_3_/150 nm-thick BaTiO_3_ system shows the capacitance boost effect with no FE-like hysteresis behavior, which was inconsistent with the experimental results; the amorphous-Al_2_O_3_/epitaxial-BaTiO_3_ system showed a typical FE-like hysteresis loop in the polarization – voltage test. This was due to the involvement of the trapped charges at the DE/FE interface, originating from the very high field across the thin Al_2_O_3_ layer when the BaTiO_3_ layer played a role as the NC layer. Therefore, the NC effect in the Al_2_O_3_/BaTiO_3_ system was frustrated by the involvement of reversible interface charge; the highly stored charge by the NC effect of the BaTiO_3_ during the charging period could not be retrieved during the discharging process because integral part of the polarization charge was retained within the system as a remanent polarization.

The high capacitance capacitor is essential for many electronic devices, including computers, digital televisions, cell phones, and electric vehicles^1^,^2^. Formerly, the dielectric thickness was decreased and higher dielectric constant materials were used to achieve a larger capacitance^3^. These conventional strategies can be well acknowledged by the extremely thin, high dielectric constant layer in dynamic random access memory capacitors and high performance logic transistors^4^,^5^. However, this approach is no longer compatible with the extreme miniaturization and lower operation voltage trends found in futuristic electronic devices and, therefore, an alternative, yet fundamentally disparate, method is necessary. The negative capacitance (NC) effect found in ferroelectrics (FE) can be an intriguing contender to solve this problem since the serial connection of NC and positive capacitance (PC), in principle, can result in an unlimited capacitance density (*C*_*total*_^*−1*^ = *C*_*PC*_^*−1*^ + *C*_*NC*_^*−1*^, meaning that total capacitance is ∞ when absolute magnitude of the NC equals to PC)^6^. This combined capacitance is not determined by the thickness or relative dielectric constant of each dielectric layer, but by the combination of dielectric thickness and relative dielectric constants of the NC and PC layers for a given area.

The capacitance is proportional to the inverse of the second order differential of internal energy (*U*) vs. electric polarization (*P*) curve of a dielectric (DE) or FE material^7^. According to the Landau’s model, the NC effect can be expected in the ferroelectrics only when the ferroelectric is unstable (polarization *P* ~ 0) with respect to the stable spontaneous polarization (*P*_*s*_) state. Therefore, NC effect from the single FE layer cannot be realized under normal situation because FE stays at the *P* = *P*_*s*_ state. Salahuddin and Datta^6^, however, suggested the possibility that the NC effect of a FE layer in a DE/FE stacked system can be stabilized when the total internal energy of the DE/FE bi-layer is minimized near *P* = 0 (also voltage *V* = 0). This could occur through the DE and FE coupling, where the polarization of each layer has an identical value, without developing domain structure. Under this circumstance, the FE layer in the DE/FE structure then shows the NC effect. Khan *et al*.^8^ recently reported that the NC effect can be observed in the hetero-epitaxial STO/Pb(Zr,Ti)O_3_ (PZT) bilayer system only when the sample was heated up to near the Curie temperature (*T*_*c*_) of PZT. Pb-based ferroelectrics generally show stronger FE property compared with Ba-based ferroelectrics in a sense that *T*_*c*_ of the Ba-based ferroelectrics is substantially lower than that of the Pb-based ferroelectrics. Based on this idea, Appleby *et al*.^9^ recently reported an experimental verification of the NC effect at room temperature from the STO/BaTiO_3_ (BTO) hetero-epitaxial layer. Furthermore, Gao *et al*.^10^ also reported a similar NC effect at room temperature from the LaAlO_3_/(Ba,Sr)TiO_3_ (LAO/BSTO) superlattice structure. The alloying of STO with BTO to make the BSTO would further lower the *T*_*C*_ which facilitates the emergence of the NC effect. Up to now, the NC effect from the DE/FE bilayer structure has been explained based on the linear combination of the free energies with respect to polarization of DE and FE layers, which were described by the phenomenological expression of Landau-Ginzburg-Devonshire (LGD)^11^,^12^,^13^. However, there were conceptual difficulties in using the displacement equation within the bilayer structure^8^,^9^. The detailed discussions on this aspect of the former energy description in the DE/FE structure could be found in on-line supplementary information (on-line SI). Another difficulty regarding the LGD theory in DE/FE system is as follows. In principle, the DE layer does not necessarily have the perovskite structures, such as STO, but could be a normal dielectric material, such as amorphous Al_2_O_3_, of which the dielectric constants are generally much lower than that of the perovskites. The LGD parameters for these materials are not very well-known, making the application of LGD theory to these cases difficult. In the case of the low dielectric DE, such as Al_2_O_3_, and high dielectric FE, such as BTO, are stacked, the electric field must be applied very unevenly over the stacked structure. Under a given external bias voltage, the FE layer in the DE/FE stack structure may play a role as the NC layer, which induces a voltage across the DE layer whose magnitude is even higher than the applied voltage (voltage boosting effect). This might induce unwanted problems, such as charge injection across the thin DE layer, which has not been considered in the previous NC effect model. When such charge injection occurs and the injected charges are trapped at the DE/FE interface, stable *P*_*s*_ can be developed within the FE layer which largely mitigates the NC effect of the FE layer.

In this work, therefore, the authors suggest an alternative approach to the possible NC effect in general DE/FE bilayer structures adopting the depolarization theory^14^,^15^,^16^,^17^,^18^. In this model, the well-known LGD theory is extended to encompass the case where the high depolarization field is present due to an imperfect polarization compensation by the interposed thin DE layer between the FE and the metal electrode. Based on this model, it was proved that the depolarization state corresponds to the aforementioned NC condition. When an external bias voltage is applied to the DE/FE system to polarize the FE layer, the FE bound charge of the FE layer at the interface between the FE layer and metal electrode can be fluently compensated by free carriers in the metal electrode. However, the FE bound charge at the DE/FE interface cannot be fully compensated by the presence of the DE layer between the FE layer and the opposite metal electrode. This induces depolarization field across the FE layer, and if the FE film is thin enough, the direction of overall field across the FE layer can be opposite to the applied field direction. In order to make the total applied voltage over the DE/FE layer equal to the external voltage, a voltage which is even higher than the applied voltage must be applied to the DE layer. This corresponds to the NC effect, i.e. voltage boosting effect, leading to the capacitance boosting effect.

This work also discusses the conditions that have hindered the operation of the NC effect in general DE/FE systems and it is the charge injection across the thin DE layer during voltage sweep. As shown in the next section, the trapped charges can largely mitigate the depolarization effect, leading to decrease in the voltage and capacitance boosting. Even more complicated problem is that the sign of the trapped charges can be reversely changed according to the polarity of the applied bias of which the magnitude is large enough to induce tunneling of carriers through the thin DE layer. Under this circumstance, the *P*_*s*_ of the FE layer can be reversibly switched as it is the case for a single layer FE, which may correspond to the frustration of the NC effect in the DE/FE layer.

Cano and Jimenez have indicated that the formation of multidomain structure, which is assumed to correspond to the depolarized state of the FE layer, can drastically decrease the probability of involving the NC effect in the structure^19^. It might be probable that FE switching involving the multidomain structure in the DE/FE system occurs when the system has high density of defect sites or embryos for the nucleation of reverse domains, where the minimization of the depolarization effect can be achieved through the closure domain pattern formation^20^. Under these circumstances, the NC effect cannot be induced because the system always stays at the minimum energy state (two positive curvature regions of the *U-P* curve of the FE layer). However, it can be anticipated that such effect is minimized in a high quality epitaxial BTO thin film, which was adopted in this work. Therefore, such possibility, i.e. FE switching mediated by the reverse domain nucleation and growth, is not taken into consideration in this work.

## Results and Discussion

### Depolarization theory of DE/FE system

**Figure 1:**
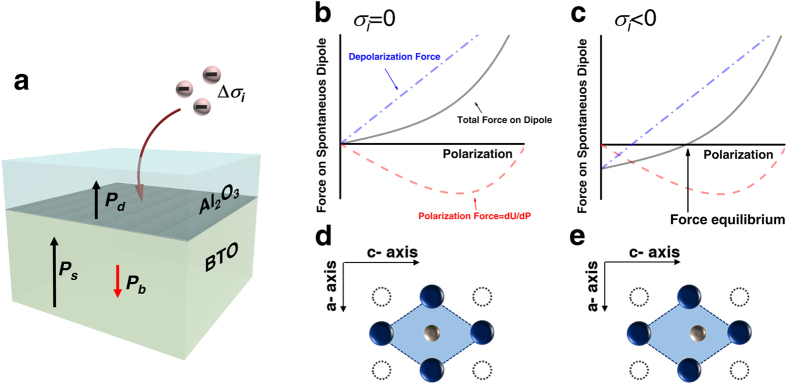
An NC model in DE/FE bilayer structure. (**a**) schematic diagram of NC model in Al_2_O_3_/BTO bilayer structure, (**b**,**c**) force landscapes of DE/FE where *σ*_*i*_ = 0 and *σ*_*i*_ < 0, (**d**,**e**) stable *P*_*s*_ configurations where the system is in (**b**,**c**) states, respectively.

In a typical metal/ferroelectric/metal (MFM) system, the net polarization charge on the FE layer surfaces generally can easily be compensated by the free carriers in the nearby metal electrodes, although it cannot be fully compensated due to the finite screening length of normal metals^14^. Almost no involvement of such adverse effect has been theoretically expected from the Pt/BTO interface^21^,^22^, which is very different from normal occasions. For the case of metal-insulator-ferroelectric-metal (MIFM) system, which corresponds to the DE/FE stack system in this work, charge compensation at the insulator side of the ferroelectric interface is hindered due to the presence of an insulator layer (DE layer). Hence, a large depolarization field across the FE layer is developed which destabilizes spontaneous polarization^14^. Figure 1a shows the schematic diagram of a general DE/FE structure. When depolarization field (*E*_*dep*_) is developed within the FE layer, it influences not only the spontaneous polarization (order parameter of Landau equation, which is sometimes called orientation polarization) but also the rest part of the FE materials, which can be considered as background polarization (*P*_*b*_). In other words, the displacement of the FE layer could be divided into two different components: spontaneous and background displacement. In contrast, there is no *P*_*s*_ in the DE layer, and thus, the equation of the displacement continuity at the DE/FE interface under the short circuit condition with or without external bias voltage should be written as equation (1),


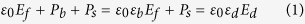


where *ε*_*0*_ represents the vacuum permittivity; *E*_*f*_ (*E*_*d*_) is the electric field inside the FE (DE) layer; *ε*_*b*_is the background dielectric constant of the FE layer; and *ε*_*d*_ is the dielectric constant of the DE layer. While the presence of *ε*_*b*_ is widely accepted in electrostatic calculations of critical phenomenon^23^,^24^, depolarization^14^,^15^,^16^,^17^,^18^, and dielectric response of the DE/FE superlattice structures^25^,^26^,^27^, the precise definition and its value are controversial. From literatures, various *ε*_*b*_ values, such as optical dielectric constant (~5)^14^,^15^,^25^,^28^, ~10^18^,^29^, ~50^22^,^30^ and >100^17^,^31^, could be found for various perovskite FE materials. In this work, 50 were taken for the *ε*_*b*_ of the c-axis oriented BTO epi-layer to calculate the thermodynamic states and the dielectric response of the FE single layer as well as the DE/FE stacked layer. Actually, *ε*_*b*_ of BTO varies according to the electric field because it could vary with variation of *P*_*s*_ along the applied field direction. However, this is generally the case for randomly oriented material, where its *P*_*s*_ state is heavily dependent on the applied field. In this work, where the epitaxial BTO film is c-axis oriented and its c-axis lattice parameter is even elongated along the surface-normal direction, the *P*_*s*_ is always aligned along the out-of-plane direction. This makes the *ε*_*b*_ quite invariant throughout the most part of voltage application. There could be bias conditions where the permittivity increases when the material is depolarized near the coercive voltage region. In fact, the depolarized state variation is quite small in one case (Fig. S3 of on-line supplementary information of ref. 32). but could be as three times high as the polarized state^33^. However, such voltage region is very narrow compared with the entire tested voltage region. Hence, a constant permittivity assumption in the calculation induced minimum error. In many theoretical cases^14^,^15^,^17^,^18^,^20^,^22^,^25^,^28^,^29^,^30^,^31^ this value has been taken as constant.

As can be understood from the equation (1), the displacement in the DE layer could be induced by only the *ε*_*0*_*ε*_*d*_*E*_*d*_ term. Therefore, if *ε*_*0*_*ε*_*d*_ of the DE layer is much smaller than the capacitive contribution from *ε*_*0*_*ε*_*b*_ and *P*_*s*_, *E*_*d*_ becomes very high. When the DE layer is very thin, interface charge (*σ*_*i*_) can be formed at the DE/FE interface by carrier injection across the DE layer. Under this circumstance *E*_*dep*_ in the FE layer decreases, which can stabilize *P*_*s*_. The electric field generated by the presence of *P*_*s*_ and *σ*_*i*_ at the FE and DE layer under the short circuit condition can be represented as follows,


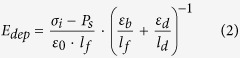



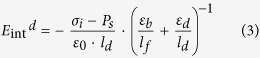


where *E*_*int*_^*d*^ is the internal electric field across the DE layer, and *l*_*f*_(*l*_*d*_) is the thickness of the FE (DE) layer. Figure 1a shows the distribution of *E*_*dep*_ and *E*_*int*_^*d*^ within the Al_2_O_3_ (AO)/BTO stacked layer, where the BTO layer possesses *P*_*s*_ and the AO/BTO interface contains *σ*_*i*_.

The free energy (or thermodynamic potential) of an order parameter (*P*_*s*_) in a FE layer can be described by LGD equation as shown in equation (4).


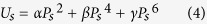


Then, the electrostatic field acting on *P*_*s*_, can be expressed as


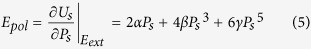


where *α, β* and *γ* are Landau coefficients of a FE material. Figure 1b,c show the electric force-polarization diagrams for the cases of *σ*_i_ = 0 and *σ*_i_ < 0, respectively. Here, the electric force can be easily calculated by multiplying the electric field with charge. The reason why a electric force is invoked can be easily understood from Fig. 1d,e; when the two forces acting on the central cation in the oxygen octahedron balance each other, the cation remains at the center position making the material paraelectric (FE polarization is completely frustrated), whereas if polarization force overcomes the depolarization force, by the presence of *σ*_i_, the material can show the FE polarization. This depolarization behavior is well established by a number of studies, especially dead layer effects in MFM thin film capacitor (See ref. 14. for example). When the FE film is thinner than the critical thickness, it becomes paraelectric which corresponds to the circumstance represented by Fig. 1b,d. On the other hand, even when the film is thinner than the critical thickness, the presence of *σ*_*i*_ at the interface could shift the force equilibrium point from the origin and *P*_*s*_ can be stabilized depending on the different values of *σ*_*i*_.

### Alternative negative capacitance model for DE/FE system

The LGD free energy equation of a FE layer under the presence of *E*_*dep*_ can be obtained by integrating the equations (2) and (5) with respect to *P*_*s*_. For the sake of simplicity, the uniform polarization in ferroelectric materials is assumed. Then, the LGD equation of FE materials with a homogeneous polarization (*P*_*s*_) and a constant *σ*_i_ under an external electric field (*E*_*ext*_^*f*^) can be expressed as


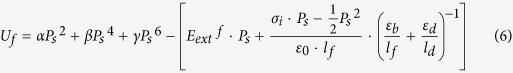


Here, the *E*_*ext*_^*f*^ is the portion of *E*_*ext*_ applied over the FE layer. The coefficient of *P*_*s*_^2^ term (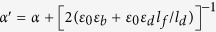) is determined by the relative magnitude of *E*_*dep*_ and *E*_*pol*_. If *E*_*pol*_ > *E*_*dep*_, *α*′ has a negative value and the FE layer is in the FE state. If *E*_*pol*_ < *E*_*dep*_, *α*′ becomes positive, and the FE layer becomes paraelectric-like. This state is critical for the emergence of the NC effect from the DE/FE structure. Detailed material parameters in equation (6) for the AO/BTO system are summarized in table 1 (Note: the ferroelastic energy had been also taken into account in energy calculations, see the equation (8).). It should be noted that the equation (6) represents the thermodynamic energy function for a given *σ*_i_. If *σ*_i_ varies, the function needs to be rewritten for new *σ*_i_, and the transition states between different values of *σ*_i_ cannot be thermodynam_i_cally described by this method. Therefore, only the thermodynamic states before and after the *σ*_i_ change are described analytically in this work, and the transition between them is only empirically described.

Then the capacitance of the paraelectric-like DE/FE system could be achieved from the general definition of capacitance. In fact, for DE/FE system, there are two distinctive layers, so the capacitance can be calculated from either layers. The derivation processes are described in detail in method section based on displacement-continuitiy at the interface between DE and FE layers and minimization of electrostatic energy. Capacitance of the DE/FE system can be represented by equation (7).


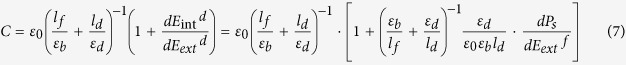


**Figure 2:**
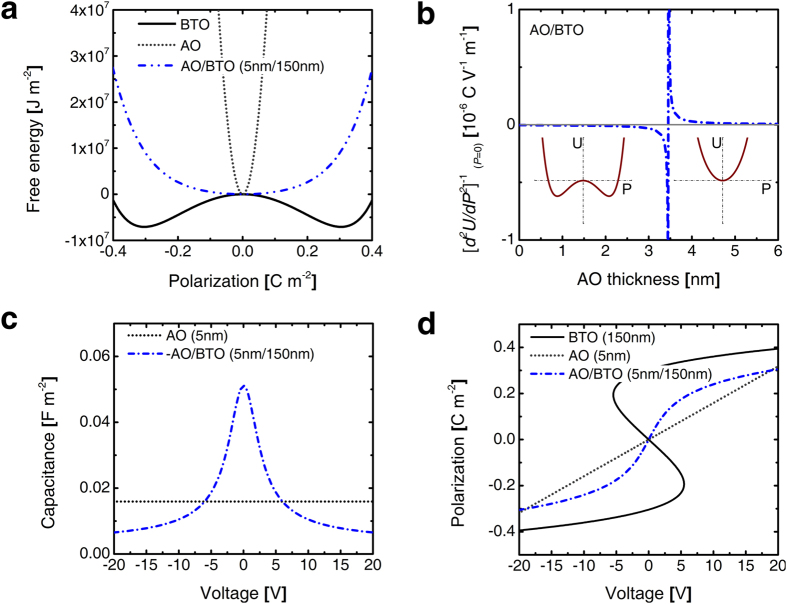
Theoretical studies on the NC effects in AO/BTO bilayer system. (**a**) Landau free energy diagrams of BTO layer in 5 nm-AO/150 nm BTO heterostructure. (**b**) AO thickness dependency of the curvature of U-P diagram, the inset figure represent energy landscape where curvature is negative (left) and positive (right) (**c**) capacitance-voltage curves, and (**d**) the spontaneous polarization–voltage curves of BTO layer in 5 nm-AO/150 nm BTO heterostructure.

Based on these formalisms, the electrical behavior of the AO/BTO bilayer structure was examined. First, the case with *σ*_*i*_ = 0 is considered in Fig. 2. Figure 2a shows the free energy diagram (*U*–*P* diagram) of a 5 nm-thick AO/150 nm-thick BTO bilayer structure calculated using the equation (6) at room temperature. For reference, the *U*–*P* curves of a single layer AO and BTO were also plotted. With this geometry, the *U*–*P* curve shows a single minimum at *P* = 0, suggesting that the ferroelectricity of the FE layer is totally destabilized due to the influence by the large depolarization field. This behavior is influenced by the relative thicknesses of the DE and FE layers. Figure 2b shows the graph of (d^2^*U*/d*P*^2^)^−1^ at *P* = 0, which corresponds to *α*′, as a function of AO thickness for the given BTO thickness of 150 nm calculated by the equation (6). The capacitance showed critical variations at 3.5 nm, which is called the critical thickness (*l*_*cr*_). When the AO film is thinner than the *l*_*cr*_, C has a negative value and diverges to −∞ as *l*_*cr*_ is approached. This corresponds to the unstable state of the DE/FE system near *P* = 0, so such overall negative capacitance cannot be experimentally achieved. By contrast, when the AO film is thicker than the *l*_*cr*_, C has a positive value and diverges to ∞ as *l*_*cr*_ is approached. This is a very useful stable state of the DE/FE for the capacitance boost near *l*_*cr*_, which is due to the involvement of the NC state of a FE layer. Nevertheless, there are two limitations on the use of such increased capacitance, which can be found from Fig. 2c,d. Figure 2c shows the capacitance – voltage (*C*–*V*) curve of the 5 nm-thick AO/150 nm-thick BTO bilayer. Here, *V* was calculated by multiplying the field and thickness of each layer and summing them. The *C*–*V* curve of single layer AO is also plotted within the same graph and shows a constant value which corresponds to a dielectric constant of 8.9. Within the voltage range from ~−5 V to ~5 V, the C value of the bilayer is higher than that of the AO single layer, suggesting the emergence of the NC state within the FE layer. However, at voltages outside this range, the C value decreases to lower values than the value of a single AO layer, which is due to the fact that the capacitance of the BTO layer changes from negative to positive at certain high voltages. Figure 2d shows the calculated *P-V* curves of the bilayer. For reference, *P*–*V* curves of single layer AO and BTO are also plotted. The *P*–*V* curve of the bilayer does not contain any negative slope region, suggesting that AO/BTO is overall in the PC state, whereas BTO layer shows the NC state. Usefulness (and limitation too) of the AO/BTO bilayer as the charge storage capacitor can be understood from these figures. For example, when the bilayer capacitor was biased from −6 V to +6 V, ~0.4 C m^−2^ is stored within the capacitor. In contrast, the single layer AO capacitor can store only ~0.2 C m^−2^. For the single BTO layer capacitor, it can store a much higher value of ~0.7 C m^−2^. However, when the capacitor voltage was released to 0 V, only ~0.05 C m^−2^ could be extracted from the single BTO layer capacitor because it is now with FE state, so ~0.65 C m^−2^ remains within the capacitor as a *P*_*r*_. For the single layer AO and bilayer capacitor, half of the stored charges is released by the same operation. As the applied voltage range increases, the stored (so released) charge density increases linearly for the AO capacitor and non-linearly for the bilayer capacitor, and finally the charge density values become almost identical for −20–20 V range. This can be understood from the decrease of capacitance at higher voltages for the case of bilayer capacitor while that of AO capacitor is constant over a whole range of voltage in Fig. 2c. The maximum storable charge density in the bilayer cannot be higher than the 2*P*_*s*_ of the BTO layer, which is the ultimate limitation of DE/FE systems as high capacitance capacitors. One may be curious about what would happen when the thickness of the AO layer is near *l*_*cr*_? According to Fig. 2b, the capacitance can be infinite, whereas Fig. 2c shows that the voltage range for such enhanced capacitance becomes infinitesimal. Therefore, there is an upper bound for the drivable charge density, which is ~2*P*_*s*_. Meanwhile, there is another critical side effect that mitigates the emergence of the capacitance boosting effect as shown in the next section.

### Formation and influence of *σ*_*i*_ on DE/FE system

**Figure 3:**
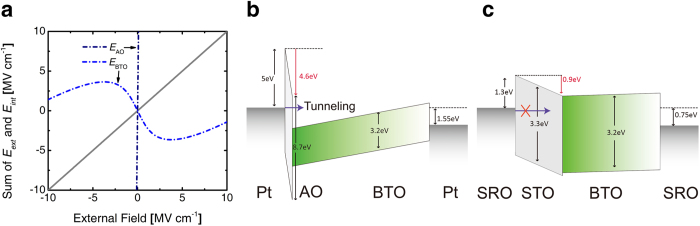
The potential distributions and the driving force for a formation of *σ*_*i*_ in DE/FE system. (**a**) external field dependence of total field (*E*_*ext*_ + *E*_*int*_) of each layer in 5 nm-AO/150 nm BTO stack structure. (**b**) band diagram of bilayer capacitor of Pt/5 nm-AO/150 nm-BTO/Pt bilayer capacitor when applying 1.55 V potential, and (**c**) SRO/25 nm-STO/50 nm-BTO/SRO bilayer capacitor when applying 0.75 V potential.

Figure 3a shows the variations in the electric field (See the equation (22) in method section) over the AO and BTO layers as a function of *E*_*ext*_ when the field was applied over the 5 nm-thick AO/150 nm-thick BTO structure with *σ*_*i*_ = 0. In this graph, *E*_*ext*_ was simply calculated by dividing the applied voltage (*V*_*app*_) by a total film thickness (155 nm). For the actual field calculation in each layer, the *E*_*ext*_ was divided into two parts: *E*_*ext*_^*f*^ and *E*_*ext*_^*d*^, which are inversely proportional to the dielectric constant of each layer (50 for BTO and 8.9 for AO), and *E*_*dep*_, calculated from the equation (2), was added to estimate the net electric field over the BTO layer. Similarly, *E*_*int*_^*d*^ was calculated from the equation (3), which is determined by the field exerted by the spontaneous polarization from the FE layer and interface charge density. *E*_*int*_^*d*^ was added to the component of *E*_*ext*_ over the AO layer. It is quite notable that the internal field across the BTO layer decreases when *E*_*ext*_ increases within −2.5 MV cm^−1^ < *E*_*ext*_ < +2.5 MV cm^−1^, meaning that the BTO layer works as a NC layer under this circumstance. Such decrease in the internal field of the BTO layer is compensated by the increase of the internal field across the AO layer, meaning that the capacitance boosting might be acquired. This outcome is obvious from the NC operation of the BTO layers. It is also notable that the field over the AO layer is extremely high even for the quite small *E*_*ext*_, which significantly influences the charge distribution as will be discussed below.

**Figure 4:**
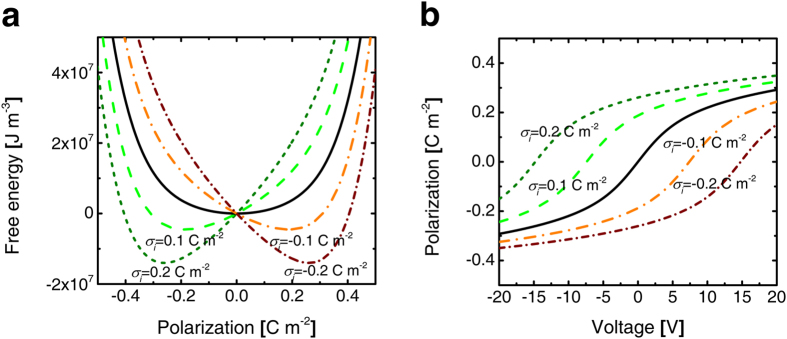
An influence of *σ*_*i*_ on DE/FE bilayer system. **a**) Landau free energy diagrams and (**b**) polarization-voltage functions of 5 nm-AO/150 nm BTO stack structure with various *σ*_*i*_ values at the DE/FE interface.

**Figure 5:**
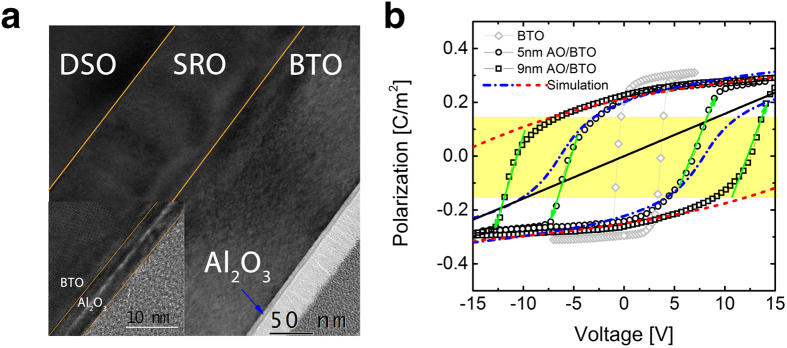
An experimental demonstration of NC model in AO/BTO bilayer structure. (**a**) bright field TEM images of the cross section of the 5 nm-AO/BTO/SRO/DSO under illumination of a 200 kV electron beam. The inset figure is a high resolution TEM image of AO/BTO interface (**b**) P-V hysteresis loops of Pt/150 nm-BTO/100 nm-SrRuO_3_ (diamond symbol), Pt/5 nm-AO/150 nm-BTO/100 nm-SrRuO_3_ (circle symbol) and Pt/9 nm-AO/150 nm-BTO/100 nm- SrRuO_3_ (square symbol) capacitors. The dash-dot blue and red dash lines represent the simulated P-V curves for 5 nm and 9 nm AO/BTO bilayer, respectively. The black line represents a P-V response of 5 nm-AO single layer. Yellow background area corresponds to the capacitance augmented region.

Figure 3b shows a schematic energy band diagram of the Pt/5 nm-thick AO/150 nm-thick BTO/Pt capacitor when *E*_*ext*_ = 100 kV cm^−1^ was applied to total structure (*V*_*app*_ = 1.55 V). Due to the NC effect of BTO under this bias condition, the BTO band is tilted in the opposite way to the applied bias direction which is compensated by the very high tilting of the AO band in accordance with the applied bias direction. The opposite tilting of the BTO band within the NC region occurs because *E*_*ext*_^*f*^ is overcompensated by *E*_*dep*_. Under this circumstance, a deep potential well is formed at the AO/BTO interface of which the depth is deeper than the conduction band offset at the Pt/AO interface. Due to a very high band tilting of the AO layer, in addition to its small thickness (5 nm), there must be a very high chance of carrier injection (by most probably tunneling) as represented by the lateral arrows in Fig. 3b. When such carrier injection occurs, the *σ*_*i*_ can compensate for the polarization charge within the BTO layer totally or partially at the interface, and *E*_*dep*_ will be diminished. This means that the NC effect could be also diminished under this circumstance. Nevertheless, it has to be noted that the influence of *σ*_*i*_ on the NC operation of the BTO layer is dependent on the bias application and carrier transport across the AO layer as discussed below. An approximate time estimation of the charge transport across the 5 nm-thick AO layer by the Fowler-Nordheim tunneling to compensate about 0.2 C m^−2^ varies from several *μs* to several tens *μs* depending on the bias voltage magnitude and other interface conditions. The influence of such carrier injection effect will be discussed in detail with Figs 4 and 5.

By contrast, it could be quite different for the case of the STO/BTO as shown in Fig. 3c. The band diagram was calculated for SrRuO_3_ (SRO)/25 nm-thick STO/50 nm-thick BTO/SRO structure. Due to a non-linear dielectric response of the paraelectric STO, the calculation was performed using a self-consistence method based on the LGD equation of STO. When *E*_*ext*_ = 100 kV cm^−1^, the calculated *E*_*int*_^*d*^ of the STO layer was 364 kV cm^−1^ and relative dielectric constants of STO layer were calculated as 215. Due to the relatively high dielectric constant of the STO, the internal band tilting of the STO layer (~0.9 eV) was much lower than that of the AO layer under the identical *E*_*ext*_ condition. The BTO band also tilts in the opposite direction to the bias voltage suggesting that the BTO layer is in NC mode. Under this band configuration, the electron tunneling from the SRO into the DE/FE interface is not expected to be active, and thus, the chance for observing the NC effect from this structure would be high^9^.

This carrier injection does not necessarily correspond to the total elimination of the NC effect as long as the value of *σ*_*i*_ is invariant during the subsequent bias application. Here, the *U-P* and *P-V* curves of the previously mentioned AO/BTO structure were formulated again based on the equation (6) assuming *σ*_*i*_, present at the AO/BTO interface, to be −0.2, −0.1, 0, 0.1, and 0.2 C m^−2^, which stayed invariant throughout the entire voltage application. The results are shown in Fig. 4a,b. As can be understood from Fig. 4a, the structure shows certain monostable polarization values corresponding to a minimum energy, with different *σ*_*i*_ values. Figure 4b shows that the *P-V* curves are non-hysteretic due to the monostable configuration of polarization under these circumstances, and the capacitance enhancement could be achieved for all cases. However, the voltage region to observe the NC operation (region with steep slope in the *P-V* curve) varies according to the *σ*_*i*_ values. The presence of *σ*_*i*_ induced an invariant internal field inside the structure and shifted the *P-V* response along the voltage direction. Therefore, the NC effect is achieved at a shifted *V* (or *E*_*ext*_) without a hysteretic *P-V* switching behavior according to this internal field effect. This can be qualitatively understood as that the invariant *σ*_*i*_ stabilizes only one of the two possible *P*_*s*_’s of the BTO layer, and this stabilized *P*_*s*_ decreases uniformly making the BTO layer be within the NC region when a bias, whose polarity is opposite to this stabilized *P*_*s*_, is applied to the AO/BTO structure. Nonetheless, as discussed previously, the change of *σ*_*i*_ along with the change in the bias polarity largely decreases the amount of retrievable charge at the discharging step, hindering the NC effect. Experimental proof of such NC frustration is demonstrated in the next section.

### Experimental study on NC effects in AO/BTO

In order to investigate NC effects in DE/FE bilayer structure, AO/BTO bilayer thin film capacitors were fabricated. Figure 5a shows the low magnification cross-section transmission electron microscopy (TEM) image of the 5 nm-thick AO grown by an atomic layer deposition (ALD)/ 150 nm-thick BTO grown by pulsed laser deposition (PLD) on the SRO bottom electrode. Inset figure shows the high-resolution TEM (HRTEM) image of the AO/BTO interface region, showing the very sharp and well-defined interface structure. From this HRTEM image, the lattice-parameter along the c-axis (normal to the film surface) was determined to be ~0.408 nm which corresponds to an in-plane misfit strain of ~−1.2% with SRO bottom layer. More detailed structural characterization of this bilayer structure can be observed in on-line SI.

Figure 5b shows the experimental *P-V* curves of a single layer BTO film with a Pt top electrode (diamond symbol) and AO/BTO bilayer film with two different AO thickness (circle symbol for 5 nm and square symbol for 9  nm). The severe shift of the *P-V* curve of the BTO single layer into a positive bias direction is due to the work function mismatch between Pt and SRO electrodes with possible contribution of epitaxial strain. Being different from calculation results shown in Fig. 4b, the *P-V* curve of the AO/BTO bilayer shows clear emergence of (distorted) hysteresis curve for the two AO thicknesses. This phenomenon can be understood from the following calculation. From equation (6), *P-V* curve of this system can be simulated. Because of epitaxial strain, modified LGD equation was adopted to correctly describe the *U-P* relationship as equation (8), which takes into account the ferroelastic energy terms^34^,


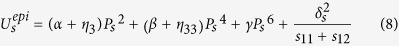


where 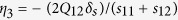, 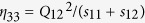, and *Q*_12_, *δ*_s_, *s*_11+_*s*_12_, are −0.034 m^4^ C^−2^ 34, −0.012 and 6.4 × 10^−12^ m^2^ N^−1^ 35, respectively. The upper and lower branches of the P-V hysteresis loop was fitted using the aforementioned formalisms under the assumption that the AO/BTO interface contains *σ*_*i*_ values of 0.080 (0.128) C m^−2^ and −0.120 (−0.155) C m^−2^ for 5 nm (9 nm) AO thickness, and the results are represented by the dash-dot lines in the Fig. 5b. The simulation fits the experimental *P-V* curve quite well in the voltage region of ~−1 V–10 V for upper branch, and of −10 V–1 V for lower branch, which means that the *σ*_*i*_ barely changed within each voltage region. The significant mismatch outside these voltage regions and transition between the two branches can be understood from the variations in *σ*_*i*_ according to the bias voltage application. For example, the *P-V* curve of the lower branch corresponds to the case where the negative *σ*_*i*_ (−0.120 C m^−2^) is stabilized mostly in the negative bias region. However, as the voltage increased into the positive bias region, *σ*_*i*_ changes to positive value most probably by tunneling through the thin AO layer (indicated by an upward green arrow in the figure). As a result, when the bias voltage reaches to +10 V it becomes 0.080 C m^−2^. With decreasing bias voltage, this positive interface charge appears to be retained down to ~−1 V but changes back to the negative value also by another tunneling process (indicated by downward green arrows in the figure). Being compared with the *P-V* curve of a single layer AO (indicated by a black line in the figure), the P-V curves of the 5 nm-AO/BTO bilayer showed a higher slope especially in the *P* region with yellow background, suggesting that the capacitance of the double layer is higher than that of the single layer AO. This corresponds to the NC operation of a BTO layer.

Nevertheless, the consecutive change in *σ*_*i*_ occurring in this region makes it impossible to observe the desired NC effect in the AO/BTO structure. The capacitance enhancement could have been achieved if charging-discharging process follows the trajectory of (non-hysteretic) *P*-*V* curve within NC region. However, the change of *σ*_*i*_ during the high voltage application shifts the *P*-*V* curve from one position to another making the overall *P-V* curve shape to be ferroelectric-like hysteretic one. This means that an integral part of the stored charges during the charging step remained in the bilayer capacitor as remanent polarization as well as the interfacial charge with the opposite sign during the discharging step. It has to be noted that the largely stored charge must be discharged spontaneously with voltage decrease when the capacitance was enhanced by the NC effect of the FE layer within the DE/FE bilayer, which was not the case in this AO/BTO sample. The tunneling through the AO layer can be easily anticipated from the very high field when BTO layer operates in NC mode (Fig. 4b). The time range for the *P* estimation at each *V* value during achieving the *P-V* loop was 100 μ*s*. This time constant is long enough to induce the sufficient charge transport across the AO layer which partly compensates for the polarization switching. It could be further noted that the trapped charge density was still lower than the *P*_*s*_ value of the BTO single layer, suggesting that induced charges on the metal electrodes were partly responsible for the stabilization of the ferroelectric bound charges.

In conclusion, the capacitance boost effect in a DE/FE system by the NC effect of a FE layer, which was originally suggested by Khan^8^, could be realized under certain limited conditions, such as no FE poly-domain formation and well-balance between the thickness and material parameters of the DE and FE layers as long as the total capacitance is in the positive regime. However, the original studies had taken some problematic assumptions, which induced a self-contradictory outcome from the calculation of the DE/FE system (See on-line SI for details). In addition, when a low dielectric DE layer is adopted, its LGD formula is not generally well known making the application of the previous formalism (Landau-Khalatnikov model)^8^ to calculate the total free energy to such cases improbable. Therefore, an alternative model was suggested in this work that could calculate the capacitance at the DE/FE system based on the general theory on the depolarization effect^14^,^15^,^16^,^17^,^18^ of the FE layer when the FE bound charge is not compensated well. This approach explains the experimental results more accurately. The model was also adopted to the case where the DE/FE interface had trapped charges which could (partly) compensate for the *P*_*s*_ of the FE layer. The charge trapping could be induced by tunneling through the thin DE layer during the NC operation of the FE layer, which augmented the potential applied over the DE layer. The interfacial charging appeared to be almost inevitable when a low permittivity DE layer, such as AO, was adopted of which the thickness must be very thin to match the absolute capacitance values of the DE and FE layers. The trapped charges stabilize one of the two *P*_*s*_’s of the FE layer, which could have induced the emergence of the NC effect from the BTO layer in the AO/BTO bilayer during the subsequent voltage application with opposite bias polarity. However, when the FE layer falls within the NC region, a significant change in the trapped charge occurs making the opposite *P*_*s*_ stabilized. Therefore, a FE-like hysteretic *P-V* loop is achieved, major portion of the accumulated charges during a voltage application is retained as the remanent polarization and injected charges in the capacitor during the subsequent voltage are released. This is detrimental to use the AO/BTO capacitor as an extremely high capacitance capacitor. It is also possible that some other factors that have not been considered in this work could contribute to the detailed *P-V* behavior of the AO/BTO structure. However, the dynamically varying interfacial charge model can provide a reasonable explanation to the experimental results. Pulse-type measurement will provide another details on the switching kinetics, which will be reported elsewhere. It was also elucidated that even when the positively infinite capacitance is realized by the perfect match between the PC of DE and the NC of FE, the overall driven charge density cannot be higher than 2*P*_*s*_ of the FE layer. This is because as the capacitance increases, the voltage range for the enhanced capacitance decreases inversely proportional to the capacitance.

## Methods

### Experimental setups

The BTO layer was epitaxially grown on a SRO/DyScO_3_ single crystal substrate by a PLD, and the AO layer was deposited by an ALD, as shown in Fig. 5a. Details for the PLD processes of the BTO and SRO layers are reported elsewhere^34^, and the ALD of Al_2_O_3_ was performed using trimethylaluminum and O_3_ (with a concentration of 250 gm^−3^) as the Al-precursor and oxygen source, respectively, at a sample temperature of 250 °C. Pt top electrode with an area of 6000 μm^2^ was fabricated by a lift-off lithographic process followed by an electron-beam evaporation of 70 nm-thick Pt layer. The *P-V* measurements were performed using Aixacct TF-2000 ferroelectric tester with an AC frequency of 1 kHz in a virtual ground mode.

### Internal field calculations

The static one-dimension Maxwell equation of each layer must satisfy the following relationship;


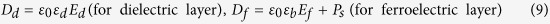


It is assumed that the polarization (*P*_*s*_) of the FE layer is homogeneous and that the trapped charges existing at the interface between the DE and FE layers fully or partly compensate the *P*_*s*_. It is further assumed that the displacements in the dielectric and ferroelectric layers 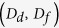 and *P*_*s*_ are constants inside each layer. Then, the Maxwell equation for each layer can be rewritten as follows from the Poisson’s equation 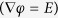;


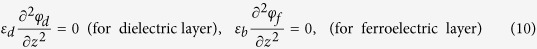


Here, the *ε*_*b*_ is the background dielectric constant of the FE layer. The boundary condition of equation (10) with a sheet interface charge density (*σ*_*i*_) at the DE/FE interface can be expressed as


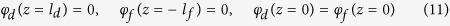


where z = 0 corresponds to the location of the interface between the DE and FE layers. By the continuity theorem at the interface, the electrical displacement and interface charge density should follow the following relationship^36^;


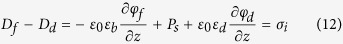



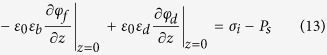


Using the geometric relationship between electric potential and static electric field,


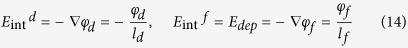


Thus, the equation (13) leads to


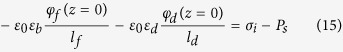


Finally, the polarization dependent internal electric field across the DE layer (*E*_*int*_^*d*^) and FE layer (*E*_*int*_^*f*^) which coincides with the depolarization field across the FE layer (*E*_*dep*_) can be obtained as follows;


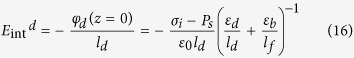



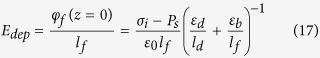


### Capacitance of a DE/FE bilayer

In MDFM (metal/dielectric/ferroelectric/metal) structure, the accumulated charge density at M/D and F/M interfaces must be identical under any arbitrary condition. Furthermore, under the absence of any interfacial trapped charges, continuity of displacement at the DE/FE interface must be maintained. Under this circumstance, the accumulated charge, *Q*, can be described either at M/D or F/M interface as follows;


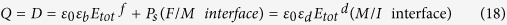


where *E*_*tot*_^*f*^ and *E*_*tot*_^*d*^ are total electric field in ferroelectric and dielectric respectively, and *ε*_*d*_ is a permittivity of an insulator. Here, the *E*_*tot*_^*f*^ and *E*_*tot*_^*d*^ encompass both electric field components from the externally applied voltage and internal charge mismatch. The capacitance of the MIFM capacitor, therefore, is


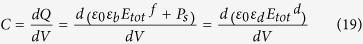


According to equation (19), capacitance of MDFM structure could be described conveniently by the charge variation at either interface. The right-hand term of equation (18) was taken to describe the capacitance here because it is more straightforward. The capacitance equation is, then, given as follows,


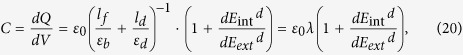


where *l*_*f*_ and *l*_*d*_ are thickness of the ferroelectric and insulator respectively. According to Kirchhoff’s law,


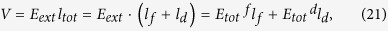


and the total electric field in each layer could be constituted by the two element as aforementioned


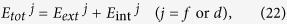


where the subscript _*ext*_ and _*int*_ represents the external and internal, respectively. Therefore,


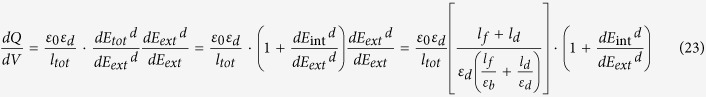


The internal field of DE layer is


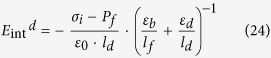


which takes into account the possible presence of an interfacial trapped charge, *σ*_*i*_. From the continuity of displacement at the DE/FE interface,


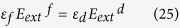


Thus, finally, equation (26) can be obtained.


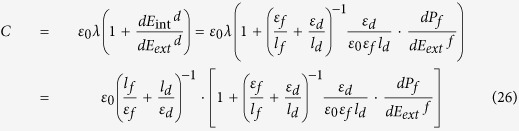


It is noteworthy that the capacitance of the MDFM structure can be simply calculated from the external field dependency of spontaneous polarization in a ferroelectric layer.

## Additional Information

**How to cite this article**: Kim, Y. J. *et al*. Frustration of Negative Capacitance in Al_2_O_3_/BaTiO_3_ Bilayer Structure. *Sci. Rep*. **6**, 19039; doi: 10.1038/srep19039 (2016).

## Supplementary Material

Supplementary Information

## Figures and Tables

**Table 1:** The material parameter for thermodynamic calculation.

Material	*T*_*c*_(K)	*ε*_*b*_/*ε*_*d*_	*α*(10^5^C^−2^·m^2^·N)	*β*(10^8^ C^−4^·m^6^·N)	*γ*(10^9^ C^−6^·m^10^·N)	Ref.
BaTiO_3_	368.5	50	3.3·(T-368.5)	1.37	2.76	37
Al_2_O_3_	—	8.9	—	—	—	—
