# Supplementary material for: Frustration of Negative Capacitance in Al2O3/BaTiO3 Bilayer Structure
Source: Sci Rep. 2016 Jan 8;6:19039. doi: 10.1038/srep19039 (PMC4705700; doi:10.1038/srep19039)
Supplement: Supplementary Information [file srep19039-s1.pdf]

Supplementary Information

Frustration of Negative Capacitance in $Al_2O_3/BaTiO_3$ Bilayer Structure

Yu Jin Kim[1], Min Hyuk Park[1], Young Hwan Lee[1], Han Joon Kim[1], Woojin Jeon[1], Taehwan Moon[1], Keum Do Kim[1], Doo Seok Jeong[2], Hiroyuki Yamada[3], and Cheol Seong Hwang[1,*]

[1]Department of Materials Science & Engineering and Inter-university Semiconductor Research Center, College of Engineering, Seoul National University, Seoul 151-744, Republic of Korea
[2]Electronic Materials Center, Korea Institute of Science and Technology, Hwarangno 14-gil 5, Seongbuk-gu, 136-791 Seoul, Republic of Korea
[3]National Institute of Advanced Industrial Science and Technology (AIST) and JST, PRESTO, Higashi 1-1-1, Tsukuba, Ibaraki 305-8562, Japan
[*]cheolsh@snu.ac.kr

I. Examnation of previous model

According to the original publication of Khan et al.[1], the following two equations constitute the basic formalism in explaining the NC effect from the DE/FE structure.

$$\varepsilon_0 E_f + P_f = \varepsilon_0 E_d + P_d \quad \textbf{(S1)}$$

$$U_{tot} = l_f(\alpha_f P_f^2 + \beta_f P_f^4 + \gamma_f P_f^6) + l_d(\alpha_d P_d^2 + \beta_d P_d^4 + \gamma_d P_d^6) - V\frac{P_f l_f + P_d l_d}{l_f + l_d} + \frac{l_f l_d (P_f - P_d)^2}{\varepsilon_0 (l_f + l_d)} \quad \textbf{(S2)}$$

ε_0 represents vacuum permittivity; α_f (α_d), β_f (β_d) and γ_f (γ_d) are the Landau coefficients of the FE (DE) material; P_f (P_d) is the polarization value of the FE (DE) capacitor; E_f (E_d) is the electric field inside the FE (DE) capacitors.

In fact, they should have used fraction, i. e., $l_f/(l_f + l_d)$ and $l_d/(l_f + l_d)$, in place of the length l_f and l_d, in equation S2 to correctly represent the total energy per unit volume of the system. So, the correct equation must be;

$$U_{tot} = \frac{l_f}{l_f + l_d}(\alpha_f P_f^2 + \beta_f P_f^4 + \gamma_f P_f^6) + \frac{l_d}{l_f + l_d}(\alpha_d P_d^2 + \beta_d P_d^4 + \gamma_d P_d^6) - E\frac{P_f l_f + P_d l_d}{l_f + l_d} + \frac{l_f l_d (P_f - P_d)^2}{\varepsilon_0 (l_f + l_d)^2} \quad \textbf{(S3)}$$

They also assumed that $P_f = P_d$, which corresponds to the strong electrostatic coupling between the DE and FE layers, which would eliminate the last term in equation S3. As a result, the external field must be applied equally to the two layers ($E_f = E_d$, equation S1).

Figure S1 shows the change in the *U-P* curves of the STO, BTO, and STO/BTO layers when an E_{ext} of 300 kV cm^{-1} was applied to the stacked layer, calculated from the previous model. The energy minimum of the each layer was achieved at P = 0.074 and 0.284 C m^{-2} for the STO and BTO layer, respectively, which is already self-contradictory to the assertion of $P = P_f = P_d$, while the overall energy minimum of the stacked layer was achieved at P = 0.169 C m^{-2}. This result shows that the equation S1 is obviously incorrect. Previous authors might have ignored the background dielectric constant of dielectric displacement of a FE layer as mentioned in main text, which caused the miscalculation. These inaccuracies resulted in errors in estimating theoretical capacitance values for the given DE/FE stack. Another conceptual difficulty related with equation S2 and S3 is that the *U* of several simpler DE layer, such as $LaAlO_3$ and Al_2O_3, of which relative dielectric constant is much lower than STO, can hardly be represented by the general Landau formula.

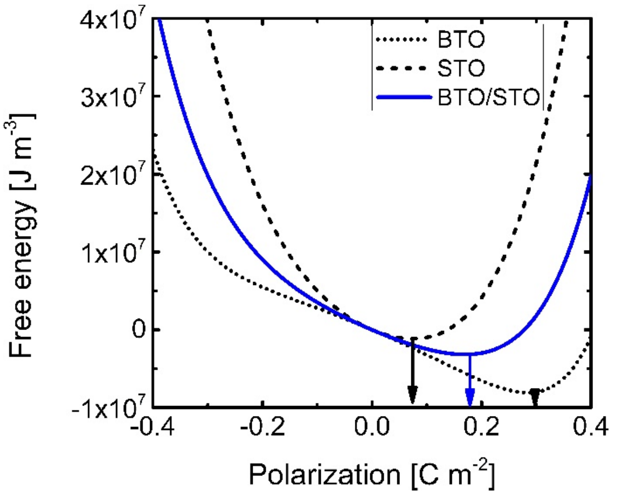

Figure S1. Landau free energy diagrams from previous model in 25nm STO/50nm BTO heterostructure under E_{ext}=300kV cm^{-1}. The dash and dot line show Landau free energy of STO and BTO single layer.The minimum positions of each energy curves represent stable polarization of each layer or structure.

II. the negative capacitance effect in STO/BTO bilayer structure.

Figure S2a shows the comparison of the calculated *U-P* diagrams of STO/BTO layers between previous model and this work under zero E_{ext}, where the thicknesses of STO and BTO layers is 25 and 50 nm, respectively. In this work, the σ_i and δ_s were taken as zero and -0.4% for epitaxially strained BTO. This work indicated a different calculation result from the original result of Ref. 2. The calculation inconsistency is believed to have occurred from the incorrect model of the previous work.[2] The *U* of bilayer, calculated in this work, is generally higher than the one calculated in the previous work for the given *P*, which resulted in the smaller overall capacitance value than the previous model. The paraelectric-like energy curves suggest that the BTO in the bilayer shows NC effect

in both models. The calculations could be extended to the cases where the BTO thickness varies for the given STO thickness (25 nm), and the results are shown in figure S2b. The experimental data from Ref. 2 are also included for comparison. Here, the capacitance data were calculated with three different δ_s value (-0.6%, -0.4% and -0.2%) at the E_{ext} = 0. All calculations expected the capacitance increase with increasing BTO thickness. This suggests that the NC effects are present, but all of the experimental data points could not be fitted with a single line in both calculations. This may be related with a strain relaxation in thicker BTO layer. The epitaxial strain generally decreases with increasing film thickness. Therefore, the fitting of experimental data with lower δ_s at higher BTO thickness in this work is a reasonable result.

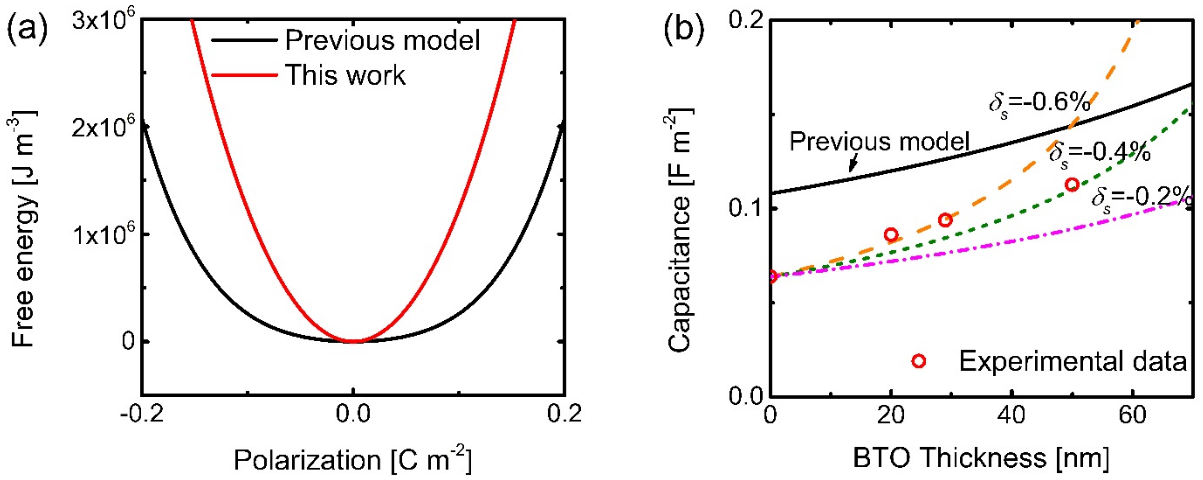

Figure S2.(a) Landau free energy diagrams of 25nm STO/50nm BTO. The red line shows the calculation results from equation 6 and 8 in main text with σ_i=0 and δ_s=-0.4%. (b) The calculated BTO thickness dependency of capacitance STO/BTO heterostructure with various δ_s. The open symbols are experimental data from Ref. 2.

III. Structural analysis of Al_2O_3/BTO/SRO/DSO thin films capacitor.

The highly epitaxial BTO ($BaTiO_3$)/ SRO ($SrRuO_3$) bilayer was grown along (001) orientation on (110) DSO ($DyScO_3$) substrate by pulsed laser deposition. Figure S3 shows the X-ray diffraction spectra of 150nm-BTO/100nm-SRO/DSO structure. It is observed that out-of-plane (c-axis) lattice parameter of BTO films was elongated due to in-plane compressive strain resulting from differences in crystal lattice parameter. Therefore, BTO film is c-axis orientated, epitaxial, and coherent with the DSO substrate, under biaxial compressive strain. The amorphous dielectric layer, Al_2O_3, was deposited by atomic layer deposition (ALD). The thickness analysis of BTO/SRO/DSO and Al_2O_3/BTO/STO/DSO layered structure was performed by X-ray reflectivity (XRR). Figure S4a and b shows the measured and the simulated XRR data for BTO/SRO/DSO and

Al_2O_3/BTO/SRO/DSO structures, respectively. The measured film thicknesses are 5nm and 150nm for $Al_2O_3$and BTO, respectively.

Figures S5a, b, and c show the bright field (BF) cross-section transmission electron microscopy (TEM) images of the cross section of the sample, where b and c correspond to the images taken after the sample was illuminated by a 200 kV electron beam for ~ 10 sec, and ~20 sec, respectively. The BF images show highly uniform and smooth interfaces between the DSO substrate and epitaxial oxide films (SRO and BTO) as well as the clean and abrupt interface between the epitaxial oxide films and amorphous Al_2O_3 film, which was highly uniform and 5.0 ± 0.3nm-thick. The SRO and BTO film thicknesses were 100 and 150 nm, respectively. An interesting observation is that the electron beam illumination induced the crystallization of the amorphous Al_2O_3 film, which can be understood from the emergence of the dark-contrast in the BF images (figures S5b and c), while the epitaxial oxide films remained intact. This is more evidently shown in Figures S5d, e, and f, which are the magnified images of figures S5a, b, and c, respectively, near the Al_2O_3 film region. Figure S5g shows the high-resolution TEM (HRTEM) image of the crystallized Al_2O_3 film by the electron beam illumination, and the inset figure shows the fast-Fourier transformation image of the crystallized Al_2O_3 film portion indicated by the box. Due to the quite rapid crystallization of the amorphous Al_2O_3 upon the electron beam irradiation, HRTEM image of the amorphous state film could not be recorded, although the amorphous structure was initially confirmed by the naked eye. The identification of the diffraction pattern revealed that the crystallized Al_2O_3 film has a γ-Al_2O_3 spinel structure[3], which has an in-plane epitaxial relationship with the underlying BTO layer. A similar epitaxial film of γ-Al_2O_3 spinel on STO was reported elsewhere[3]. Atomic layer deposited Al_2O_3 films generally have an amorphous structure, due to its low deposition temperature (250 – 300 ^{o}C) on a non-epitaxial substrate, such as Si/SiO_2, of which amorphousness could be retained after post-annealing at high temperature (up to ~ 1000 ^{o}C [4]). It appears that the epitaxial nature of the bottom layer and lattice match between the bottom oxide layer and spinel-structured Al_2O_3 induced such an unexpected solid state epitaxial phase transformation in the Al_2O_3 layer. While this is an interesting topic for further investigation, it is certainly over the scope of the present work. More importantly, however, the application of bias voltage for the electrical test will certainly not induce such solid state phase transition since the carrier transport across the Al_2O_3 must be much lower in the electrical test. Figures S5h, and i show the HRTEM images of the interface regions of DSO/SRO, and SRO/BTO, respectively. It could be understood that both interfaces are atomically flat and clear with little indication of any chemical intermixing.

Figure S6a and b show the topographic atomic force microscopy image of the BTO/SRO/DSO and Al_2O_3/BTO/SRO/DSO structures, respectively. They show almost no difference suggesting that the growth of Al_2O_3 film by the ALD process on the BTO/SRO/DSO structure induced no structural damages. The root-mean-

squared roughness values of the two samples estimated from an area of 1 x 1 μm^2 were 0.29 and 0.31 nm, respectively, suggesting highly smooth morphology and high quality of the films.

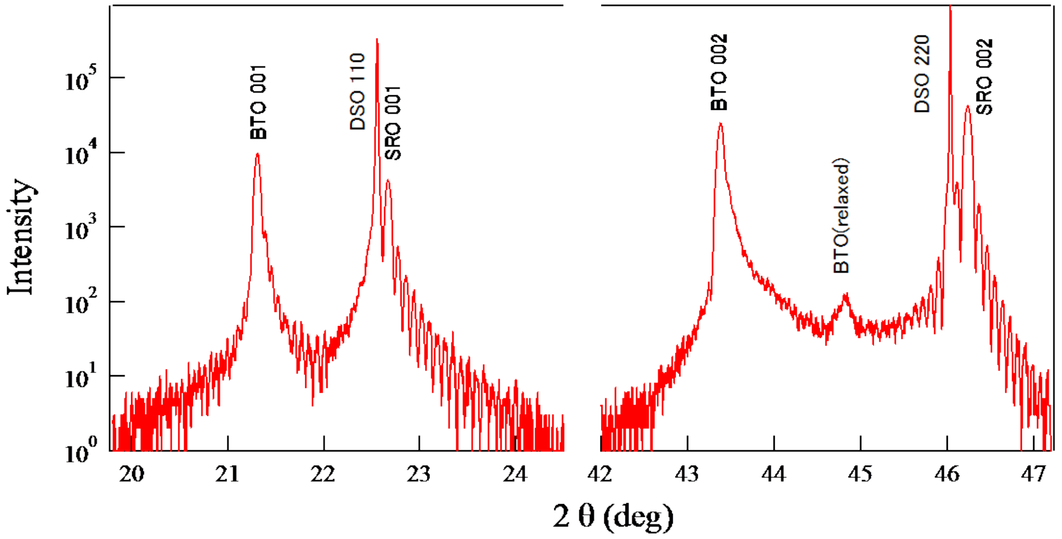

Figure S3. a XRD spectrum of 150nm-BTO/100nm-SRO/DSO epitaxial structure. Identical diffraction planes of each peak are marked in figure.

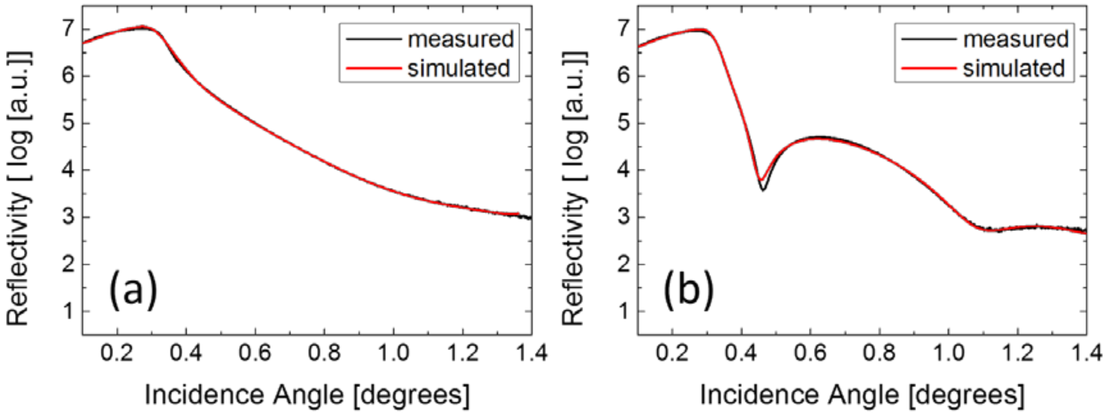

Figure S4. XRR spectra of (a) 150nm BTO/100nm SRO/DSO and (b) 5nm Al_2O_3/150nm BTO/100nm SRO/DSO.

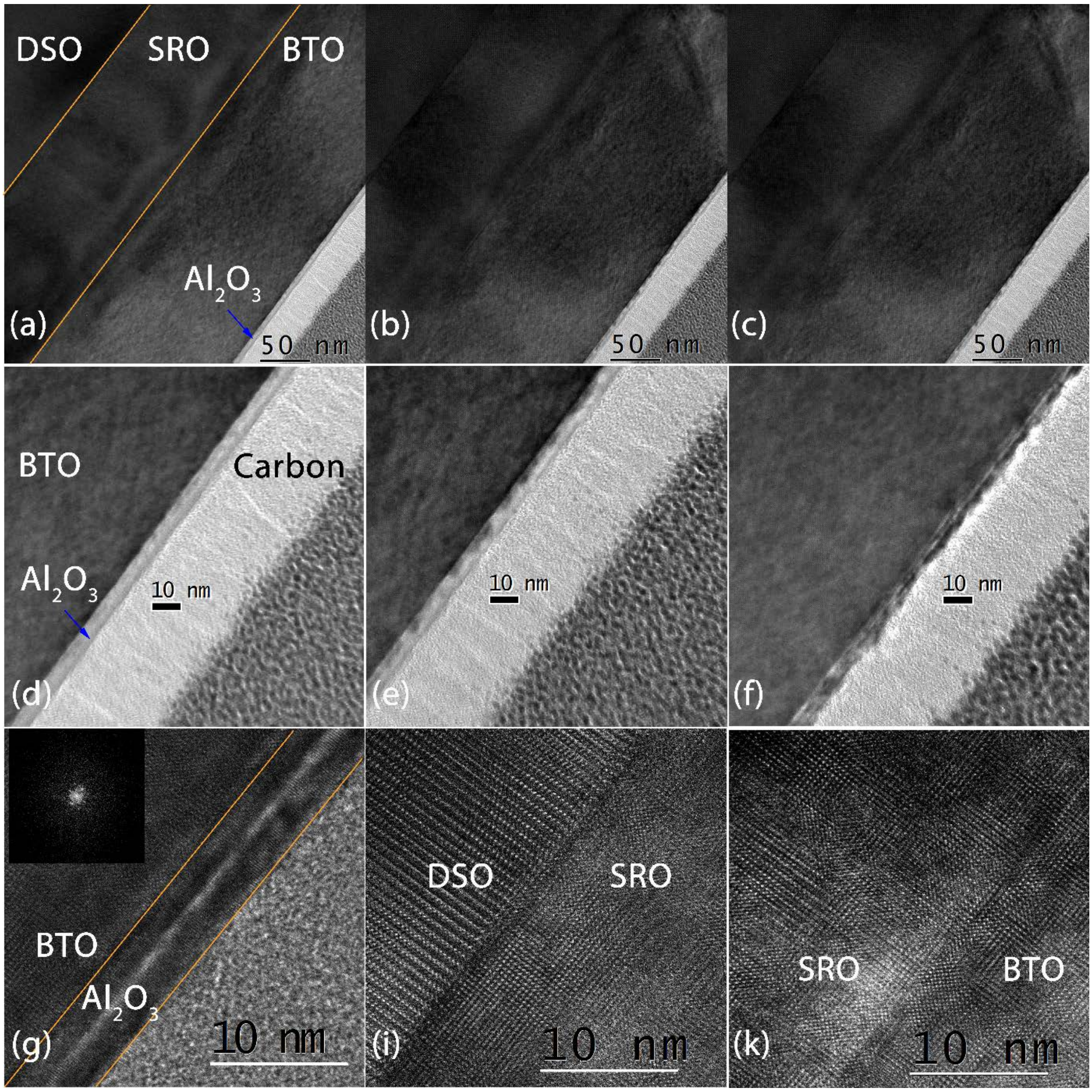

Figure S5. The bright field TEM images of the cross section of the Al_2O_3/BTO/SRO/DSO under illumination of a 200 kV electron beam for (a) 0sec (b)~ 10 sec, and(c) ~20 sec. and the (d), (e), and (f) are the magnified images of (a), (b), and (c), respectively. The High resolution TEM image of interface of (g) Al_2O_3/BTO, (i)DSO/SRO and (k)SRO/BTO. The inset figure in (g) is FFT image of Al_2O_3 layer.

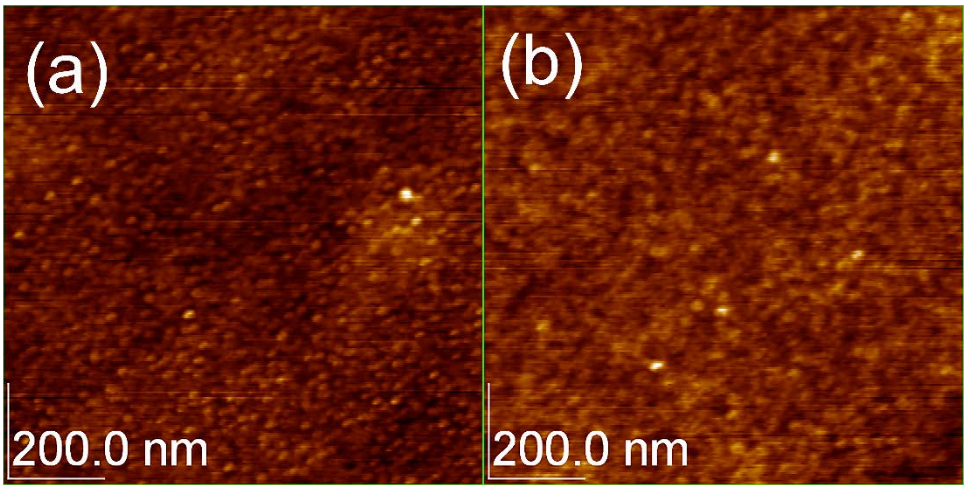

Figure S6. Topographic atomic force microscopy images of (a) the BTO/SRO/DSO and (b) Al_2O_3/BTO/SRO/DSO structures.

[1] Khan, A. I. *et al.* Experimental evidence of ferroelectric negative capacitance in nanoscale heterostructures. *Appl. Phys. Lett.* **99**, 113501 (2011).

[2] Appleby, D. J. R. *et al.* Experimental Observation of Negative Capacitance in Ferroelectrics at Room Temperature. *Nano Lett.* **14**, 3864-3868 (2014).

[3] Chen, Y. Z. *et al.* A high-mobility two-dimensional electron gas at the spinel/perovskite interface of γ-Al_2O_3/$SrTiO_3$. *Nat. Commun.* **4**, 1371 (2013).

[4] Zhang, L., Jiang, H. C., Liu, C., Dong, J. W. & Chow, P. Annealing of Al_2O_3 thin films prepared by atomic layer deposition. *J. Phys. D: Appl. Phys.* 40, 3707–3713 (2007)
